# Wythe's Pocket Dose and Symptom Book

**Published:** 1853-01

**Authors:** 


***Wythe's Pocket Dose and Symptom Book.*** Philadelphia, Lindsay and Blakiston, 1853.

A physician has no business to require such a work as this, but, while doctor-factories continue so numerous and the competition for business among them so active, books of this kind will continue to be as necessary as saddle-bags or lead-pencils. To those who need such aids, and their name is legion, we can recommend this, which appears to be carefully compiled from the best authorities. It is certainly better to carry such a little manual as this in the pocket, than to make murderous blunders in the administration of medicines.

